# Productive biosensing techniques empowered by all-dielectric metasurfaces

**DOI:** 10.3389/fbioe.2024.1484638

**Published:** 2025-01-09

**Authors:** Masanobu Iwanaga

**Affiliations:** Research Center for Electronic and Optical Materials, National Institute for Materials Science (NIMS), Tsukuba, Japan

**Keywords:** all-dielectric metasurface, fluorescence, biosensor, DNA, antibody, antigen, big data, AI

## Abstract

Artificially designed, functional nanostructured surfaces, called metasurfaces, are an emerging platform for biosensing. Two major types of metasurface biosensors have been reported: one is based on resonant-wavelength shift and the other is specialized for fluorescence (FL) detection. The all-dielectric metasurfaces that composed of periodic arrays of silicon nanocolumns have a series of optical magnetic-mode resonances, some of which were found to significantly enhance capability for FL detection of diverse target biomolecules, ranging from nucleic acid to antigens and antibodies. Here, we mainly address the recent advances in productive metasurface FL biosensors, provide an overview of the pivotal results, and discuss the future prospects, including artificial-intelligence-driven big data analysis for the next-generation healthcare services.

## 1 Introduction

Biosensing data are growing at a high rate and forming big data. To exploit these, rapidly developing artificial intelligence (AI) will play a key role. To date, the established function of AI has been to extract reliable *averaged* information from large amounts of data. As is widely known, human languages are outstanding big data, being successfully made use of in large language models of AI. Thus, for optimal use of AI in the field of biotechnology, it is crucial to collect and accumulate scientific data using reliable biosensing and/or bioimaging techniques. In this context, the biosensing techniques are expected to enable the efficient acquisition of numerous precise data on biomolecules related to living bodies.

**FIGURE 1:**
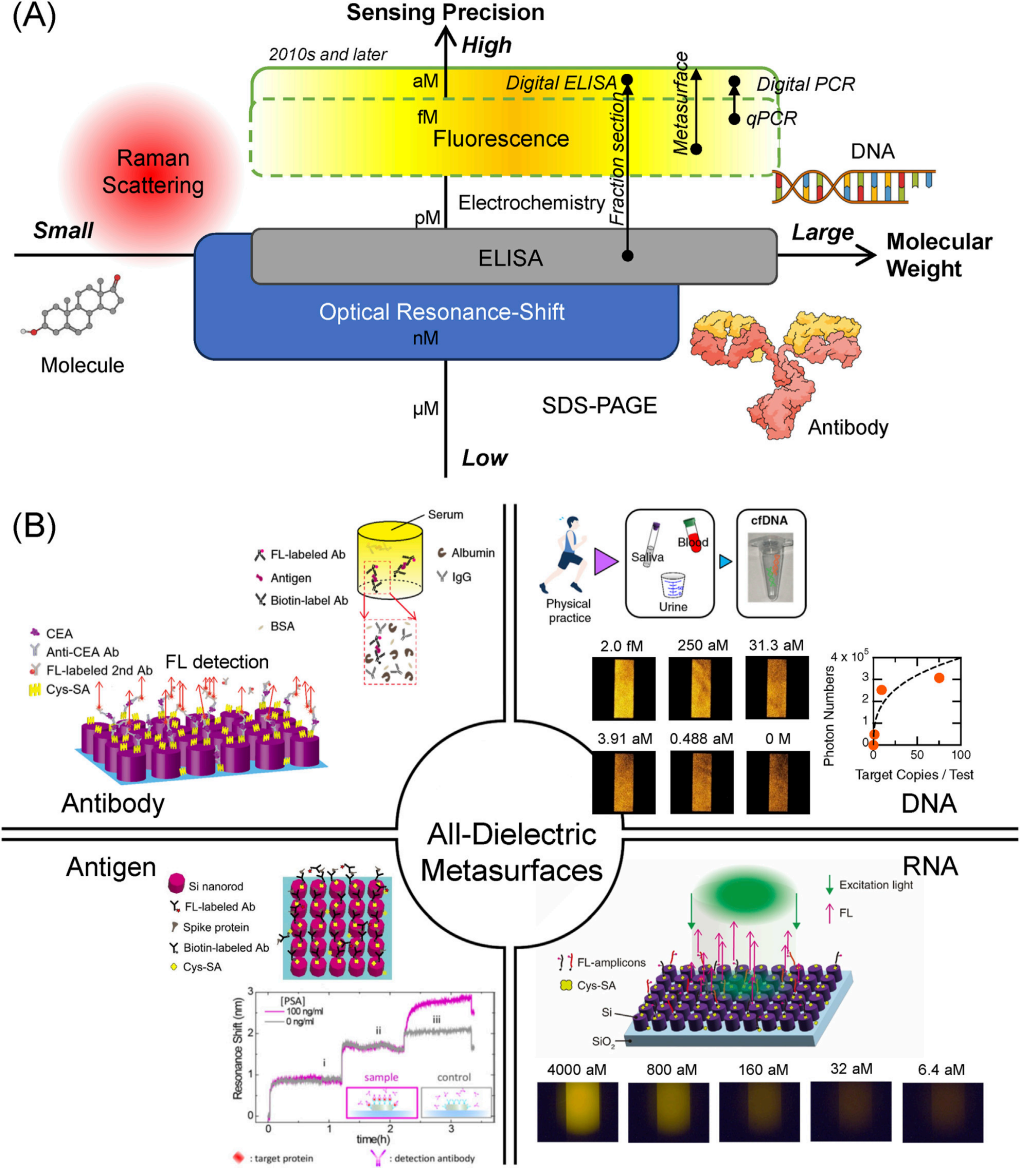
**(A)** Practical biosensing techniques classified by target molecular weight (horizontal axis) and sensing precision (vertical axis). Each biosensing technique is positioned, according to its target molecular weight and sensing precision, which is inversely proportional to LOD. The highest sensing precision is sub aM, reaching single-molecule concentration. Arrows indicate technical improvements in sensing precision with help of new technologies. Large molecules, such DNA and IgG-type antibody, and a small molecule are illustrated. **(B)** Target biomolecules detected by the all-dielectric metasurfaces to date. The targets include diverse biomolecules, ranging from proteins, such as antibody and antigen, to nucleic acids, such as DNA and RNA. Antibody is indicated using the abbreviation Ab. BSA and Cys-SA denote bovine serum albumin and cys-strepravidin, respectively. Top-right and bottom-left antibody images are cited from Iwanaga (2023b), Iwanaga (2020), respectively. In the antigen region, Spike-protein image is from Iwanaga and Tangkawsakul (2022) and PSA-detection image is from Yavas et al. (2017). Copyright (2017) American Chemical Society. DNA image is from Iwanaga et al. (2023a). RNA image is from Iwanaga (2022).

Biosensing has been studied extensively for several decades. Several biosensing techniques have been used to detect biomolecules: gel-based mass analysis such as sodium-dodecyl-sulfate poly-acrylamide gel electrophoresis (SDS-PAGE), immunoassay represented by enzyme-linked immunosorbent assay (ELISA), optical resonance shift, Raman scattering, fluorescence (FL) detection, and electrochemical techniques. According to the weight of the target biomolecules and the sensing precision, the biosensing techniques are arranged as shown in Figure 1A, which schematically illustrates the positions of each method in the plane of molecular weight and sensing precision, where the precision is defined as the amount that is inversely proportional to limit of detection (LOD) in each method. Large molecules, such as DNA and immunoglobulin G (IgG)-type antibody, are functional biomolecules in living cells and bodies. Relatively small molecules, such as sucrose and amino acids, play a role in maintaining the living systems. Qualitatively, the biosensing techniques based on the optical resonance shift, ELISA, FL detection, and electrochemical measurement are mainly used for the huge molecules, whereas Raman scattering including surface enhanced Raman scattering (SERS) is applied to small molecules to detect their vibrational signals, which are often referred to as molecular fingerprints.

To seek for higher precision in the detection techniques, the FL detection and electrochemical measurement are mostly employed in recent biosensing studies. Regarding the electrochemical sensing, it was often claimed that high sensitivity was obtained over a wide range of target biomolecule concentrations (Miao et al., 2018; Khan et al., 2018). However, the electrochemical sensors generally exhibit very low responsivity, that is, the detected signal changes are typically 20% or less for one order of target concentrations. Consequently, it is difficult to provide a precise multi-order-digit analysis for target concentrations. In contrast, FL detection yields more precise and specific results for the target concentrations, owing to the significant development of FL-protein-labeling techniques that started from the finding of green fluorescent proteins (Shimomura, 1979). This is one of the reasons that methods to detect FL are considered to be most precise and are capable of achieving the lowest LOD among the various biosensing techniques to date (Giljohann and Mirkin, 2009). Single-molecule sensing was claimed to use SERS before 2000 (Nie and Emory, 1997; Kneipp et al., 1997); however, the SERS sensing was conducted at high target concentrations, indicating that the SERS signals were observed as rare events involving many target molecules at a large LOD.

In 2010s and later, further technical improvements in FL detection were ma made, substantially advancing the sensing precision to detect target biomolecules. One of the approaches was the fraction section method (Rissin et al., 2010), which measured the FL signals in sectioned areas up to 1,000,000, conducted statistical analysis, and enabled a wide dynamic range of the FL signals. Conducting the elaborate measurement and analysis procedures, the signal-to-noise ratio was substantially improved. This sectioning method is often referred to as the digital method; for instance, digital ELISA and digital polymerase chain reaction (PCR) were commercialized. Another approach was realized by employing new FL-enhancing platforms, particularly metasurfaces (Choi et al., 2015; Iwanaga et al., 2016; Iwanaga, 2018). Incorporating microfluidic chips, the metasurfaces formed compact FL biosensors and enabled highly precise biosensing (Iwanaga, 2020). In particular, single cell-free DNA (cfDNA) was successfully detected by discriminating one cfDNA from zero cfDNA (Iwanaga et al., 2023); to the best of our knowledge, such ultimate single-molecule detection has not been attained in any other biosensing platform. These recent developments are shown in Figure 1A and listed in Supplementary Table S1.

In this paper, we mainly address the recent advances in all-dielectric metasurface biosensors. They were applied to resonance shift and FL biosensing. In Section 2, the working principles are described. In Section 3, recent advances in the metasurface FL biosensors are overviewed. In Section 4, good practices to use AI are discussed.

## 2 Biosensing techniques using metasurfaces

In this section, we briefly address sensing targets and the working principles of biosensing techniques using the all-dielectric metasurfaces. In addition, the limit of detection (LOD) in each technique is referred to.

### 2.1 Resonance-shift detection

#### 2.1.1 Detection of spectral changes

Optical resonances in metasurfaces are observed in their reflection or transmission spectra. The prominent resonances respond to changes of the outermost surface of nanostructures constituting the metasurfaces, resulting in spectral shifts of the resonance. By calibrating the shift with amount of molecules absorbed onto the outermost surface, the concentrations of target molecules can be determined. This method was initiated using surface plasmon resonance (SPR) on flat gold film in 1980s (Raether, 1988). The SPR method is now commercialized, being used as an analyzing instrument to evaluate biomolecule binding reaction (Myszka et al., 1998).

Although the SPR method is an established analytical method, the LOD is typically in the nanomolar (nM, M = mol / L) to sub-nM range. Furthermore, the optical configuration requires deep oblique incidence, which is complicated and demanding in terms of space. Therefore, further improvements in LOD and optical configurations were pursued. In 2010s, dielectric nanostructures were recognized as optical resonators (Gomez-Medina et al., 2011), which are associated with prominently resonant spectra. All-dielectric metasurfaces of silicon nanodisk arrays were used to detect a cancer marker protein, prostate-specific antigen (PSA) (Yavas et al., 2017); analysis of the transmission spectrum shift indicated that the LOD was approximately 100 picomolar (pM), comparable to the LOD of commercially available ELISA kits. The benefit of using the all-dielectric metasurfaces is the simple optical setup that allows normal incidence.

A new twist in resonance-shift sensing is to use the bound states in the continuum (BIC) in all-dielectric metasurfaces. The BIC plays a role in realizing modes with high-quality (Q) factors (or narrow linewidths), in accordance with slightly symmetry-breaking structure(s). The high-Q modes facilitate tracing resonance shift, compared to low-Q modes, in accordance with the change in the environmental refractive index or the absorption of molecules on the outermost surface of the metasurfaces. Realizing this concept, BIC-based resonance-shift sensing was reported (Hsiao et al., 2022; Watanabe and Iwanaga, 2023; Watanabe and Iwanaga, 2024). The physical limit of resonance shift sensing is given by a relation of Δλ=a×Δn where Δλ , a , and Δn denote the wavelength shift, periodic length of the metasurface, and change in environmental refractive index, respectively (Iwanaga, 2023a). The BIC-based metasurfaces showed 50%–70% realization of the physical limit. Thus, there is a limit, even if the sensitivity is increased; therefore, narrow linewidth associated with the high-Q mode is used to obtain better figure of merit (or signal-to-noise ratio) in the BIC metasurfaces. Even when these improvements are incorporated, the LOD will remain at a pM range. Thus, it is unlikely that extremely high sensitivity at an attomolar (aM) range is realized using the BIC metasurfaces.

#### 2.1.2 Detection of molecular absorption lines

Molecules have their light absorption lines in the infrared range. The absorption spectroscopy is valid for rather small molecules because large molecules with molecular weight more than 50,000 have complicated absorption spectrum and are difficult to be identified. Detection of a series of absorption lines of a particular molecule may be useful to prepare a series of metasurfaces with a broad absorption band corresponding to the molecular absorption lines. This concept was demonstrated using a set of all-dielectric metasurfaces, each of which had a narrow infrared absorption band originating from BIC in accordance with the asymmetric units (Tittl et al., 2018). Although the LOD was not stated, the shown data suggested that the LOD was in a nM range. This means that the method is valid only for high-concentration targets.

Thus, the biosensing techniques based on resonance shifts are suitable for analyses at rather higher target concentrations. In this sense, they are not highly precise sensing techniques but are characterized as analyzing techniques regarding molecular binding reaction and molecular identification at moderate and higher concentrations.

### 2.2 FL detection

Methods for efficient FL detection have been pursued for tens of years. In 1970s, it was found that FL quenching becomes dominant when FL molecules were placed on a flat metal or dielectric material (Chance et al., 1978). The effect was understood as ultrafast transfer of photoexcited states.

In the era of nanotechnology since 2000, many nanostructured surfaces on substrates were tried to improve the FL-detection efficiency; some of them showed prominent FL-enhancing effects (Kinkhabwala et al., 2009; Fu et al., 2010; Zhang et al., 2012; Zhou et al., 2012; Punj et al., 2013; Choi et al., 2014; Choi et al., 2015; Iwanaga et al., 2016; Iwanaga, 2018; Dong et al., 2021). In these studies, more than 1,000-fold FL-intensity enhancing effects were reported, where the enhancement was defined as the FL-intensity ratio of FL intensity on the nanostructures to the FL intensity on relevant flat references. Reproducibility, which is defined here as percentage to measure the best FL enhancement, was sufficiently high for only two cases: plasmo–photonic metasurfaces (Choi et al., 2015; Iwanaga et al., 2016) and all-dielectric metasurfaces that feature higher magnetic-mode resonances (Iwanaga, 2018). These two cases adopt a strategy to optimize the entire photoexcited process from excitation to FL emission. In contrast, the other cases focused on the so-called hot spots, which were electric-field enhanced local spots, emerging in nanogaps or nanocorners.

## 3 Metasurface fluorescence biosensors

Figure 1B summarizes the representative results on the detection of biomolecules using the all-dielectric metasurfaces. The targets were diverse, ranging from antigen/antibody (Section 3.1) to DNA/RNA (Section 3.2). Labeling FL probes on the antibodies or DNA probes specific to the target ensures that the FL sensing is specific to the target. The practical potential of the metasurface biosensors is also referred to (Section 3.3).

### 3.1 Antigen/antibody detection

Biosensing primarily targets proteins of diverse sizes and varieties. When focusing on biosensors for healthcare applications, antigen and antibody among the numerous proteins are suitable sensing targets. On the left side of Figure 1B, antibody and antigen detection using the all-dielectric metasurfaces of silicon circular nanocolumn array is illustrated. The typical structural parameters were as follows: periodic length of the metasurfaces was 300 nm, and the diameter and height of the nanocolumns were 220 and 200 nm, respectively. The parameters were selected considering the excitation wavelength of 532 nm and FL-emission wavelengths of 570–630 nm. A higher magnetic-mode resonance was tuned to the FL-emission wavelength (Iwanaga, 2018). Nanofabrication procedure of the metasurfaces was subjected to a top-down approach using electron-beam lithography (Iwanaga, 2020).

**FIGURE 2:**
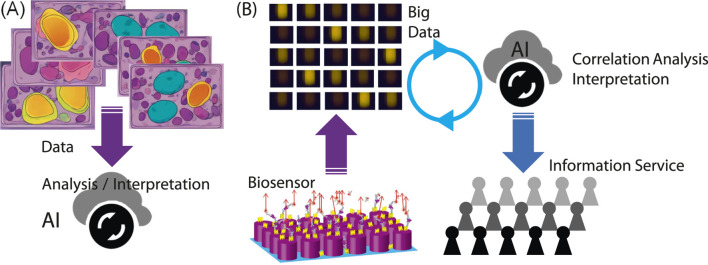
**(A)** Illustration of a commonly considered scheme to use AI in biosensing and bioimaging. **(B)** Scheme involving productive biosensors, big data, AI, and healthcare information services for people. The FL and metasurface images are adapted from Iwanaga (2022), Iwanaga (2020), respectively.

The antigen/antibody detection on the metasurfaces was conducted in sandwich configurations where an antigen was bound to the two specific antibodies, as illustrated in Figure 2. In the direct sandwich configuration, an antibody labeled with biotin and another antibody labeled with FL molecules; the biotin-labeled antibody was bound to the streptavidin (SA, represented by yellow marks in Figure 2) that were first immobilized on the metasurfaces; the FL-labeled antibodies emitted FL signals specific to the target on the metasurfaces in an FL-enhanced manner. In the indirect configuration, the FL-labeled antibodies were bound to the non-conjugated primary antibody that formed the sandwich body. The concrete microfluidic protocols were described in previous reports (Iwanaga, 2020; Iwanaga, 2023b).

The antigen/antibody detection results shown in Figure 1B are briefly described below.

(i) Cancer marker antigens, such as carcinoembryonic antigen (CEA) and PSA, were detected in a dynamic range of four orders of target concentrations, and the LOD were 10 femtomolars (fM) and 4 pM, respectively. Importantly, both CEA and PSA were successfully detected without substantial reduction of the FL signals in human serum, which is a practical medium for medical examinations (Iwanaga, 2023b). We note that the medical diagnostic criteria regarding CEA and PSA are 1,000- and 25-fold higher than the LOD, respectively. Therefore, the biosensing on the metasurfaces is sufficiently precise for the medical cancer diagnoses. Additionally, in the experiment for direct comparison, the CEA was detected using a commercial ELISA kit, and the LOD was approximate 5 pM, being 500-times higher than that by the metasurface biosensors (Iwanaga, 2023b).
(ii) The spike proteins of COVID-19 were detected and the LOD was identified to be 0.8 pg/mL, which is estimated to be approximately 292 fM. In a similar configuration, the antibodies of the spike proteins were also detected in the range of 10.7–686 pM (Iwanaga and Tangkawsakul, 2022), which can serve as a test of the antibody level. This is one of the advantages of the metasurface FL biosensors that multiple targets are detected in the same platform by adjusting the microfluidic protocols.
(iii) IgG antibody was detected in a wide range from pg/mL to tens of ng/mL, and the LOD was determined to be 34 fM. This detection capability of IgG was better than that of the commercial ELISA kits, which exhibited the LOD of 1.56 pM in the experiment for direct comparison (Iwanaga, 2020).

The all-dielectric metasurfaces have recently been applied to human serum albumin (HSA) detection in urine (Fu et al., 2024). In this experiment, the molecular configuration used for detection was different from that used for previously described sandwich assays. Instead of the ordinary FL probes labeled on antibodies, we used a material to show aggregation-induced emission (AIE) on HSA, which was termed as TPE-4TA and emitted green FL peaked at 530 nm (Tu et al., 2019). The AIE material binds to the HSA in a specific manner, induces the deformation of HSA, further aggregates on the HSA, and increases FL emission. In conventional measurements using microwells, the AIE material enabled HSA detection at 0.25–1,000 μ g/mL. Using the metasurfaces, a lower concentration range of 0.0188–160 μ g/mL was successfully measured and the LOD was identified as 18.75 ng/mL, which was more than 10-fold lower than that of the conventional measurements. Thus, the metasurface platform exhibited the precise sensing without loss of robustness for a type of realistic biopsy samples, urine.

### 3.2 Nucleic acid detection

Nucleic acid detection using all-dielectric metasurfaces has frequently been reported (Iwanaga, 2021; Iwanaga, 2022; Iwanaga et al., 2023), which are based on the same design as that of the antigen/antibody detection described in Section 3.1. In the basic detection scheme, the binding molecule SA was first immobilized on the outermost surface of silicon nanocolumns, biotin-conjugated DNA probes were efficiently immobilized on the metasurface via the biotin-streptavidin coupling, the target DNAs with a complementary sequence to those of the biotin- and FL-probes were immobilized on the metasurface, and FL measurement was conducted. An illustration of the FL detection is provided in Figure 1B. Overall, the scheme is similar to that for the sandwich protein detection, being based on target-specific detections.

There is an option that the target DNA sequence can be amplified through thermal cycling in a manner similar to PCR if the original target concentrations are too low, such as in the aM range. With help of FL enhancement of the metasurfaces, the amplification cycles are reduced, which is important for suppressing false positive reaction that could occur in ordinary PCR.

As typical results, we here refer to three results.

(i) Direct DNA detection (without amplification) resulted in DNA detection in a pM range. LOD for a target DNA was 0.1 pM (Iwanaga, 2021). This procedure was the simplest for DNA detection.
(ii) A low-concentration target of COVID-19 complementary DNA was detected in the range of aM to fM with amplification cycles shorter than those of conventional quantitative PCR (qPCR). The LOD of 5.86 aM (or 14 copies/test) is better than qPCR and equivalent to that of digital PCR (Iwanaga, 2022); typical results that reported COVID-19 RNA detections were 40 ± 10 copies/test using the qPCR (Bruce et al., 2020) and approximate 10 copies/test using the digital PCR (Yin et al., 2021). It is to be noted that thermal cycling for amplifications were different in the three cases; that is, the metasurface biosensors were used after 35 cycles, the qPCR conducted 40 cycles, and the digital PCR did 45 cycles; therefore, the ratio of amplified products is 1:32:1,024 , respectively, meaning that the metasurface biosensors were far less relied on the thermal cycle amplification; however, the metasurface biosensors exhibited the high sensitivity, thanks to the exceptional FL-enhancement capability. The short cycle operation significantly reduces falsely positive results without loss of the robustness and is one of the advantages to use the metasurface biosensors. As is widely known, the complementary DNA is directly produced via reverse transcription of RNA. The acquired data were FL images of the metasurfaces captured by a charge-coupled device (CCD) camera, as shown in Figure 1B, and analyzed in an automated manner. The data acquisition and straightforward analysis enabled us to obtain massive biosensing data. Thus, the metasurface biosensor system is a productive sensing platform.
(iii) cfDNA is a short DNA fragment of 150–200 base pairs that circulates in human blood. The cfDNAs are released from various organs and have signatures specific to diseases or reactions. They are considered as next-generation biomarkers (Cristiano et al., 2019). However, the concentration is extremely low and requires ultrahigh-precision detection techniques. Under this requirement, we applied the metasurface platform for cfDNA detection and achieved single cfDNA detection (Iwanaga et al., 2023). In particular, one cfDNA was definitely discriminated from zero cfDNA with high statistical confidence. Such an ultimately precise detection has not been shown in any other biosensing platform; for example, the digital PCR usually exhibits the LOD at approximate 10 copies/test (Hu et al., 2020; Wei et al., 2022).

### 3.3 Practical potential

We here address the metasurface FL biosensors from a practical viewpoint. The metasurface substrates and the microfluidic chips are mass productive in foundries conducting nano/microlithography. The measurement setup of metasurface biosensors has been built in a compact, cost-effective, and automated form Iwanaga et al. (2023b), enabling high-throughput operation in biosensing. Thus, the total cost is able to be suppressed, being sufficiently practical in comparison with the existing commercial biosensing techniques.

## 4 Discussion and future scope

Here, we discuss best practices for the use of AI in biosensing. Figure 2 shows two plausible uses of AI in situations related to biosensing. Figure 2A illustrates the AI involved in biosensing or bioimaging. Figure 2B shows a scheme for AI usage in a cycle of healthcare services, starting with a productive biosensor.

Owing to the rapid development of AI technologies, trials to involve AI in biosensing and/or bioimaging will increase over the next few years. Indeed, massive stacked medical images such as X-ray and magnetic resonance images are being learned and analyzed by AI in machine-learning schemes. There are already numerous images regarding ordinary medical examinations, so AI is expected to assist medical doctors in make diagnoses. This is a stereotype expectation for AI.

When considering the AI applications shown in Figure 2A to new biosensors or bioimaging devices, it is necessary to prepare a large amount of teaching data, along with accurate human knowledge. This would be a demanding task in developing a new biosensing/bioimaging application associated with AI. To avoid this demanding task, it may be helpful to link newly acquired data with the existing, known data in a reasonable manner. When building this link, AI could be a handy and cost-effective tool because the current AI has already learned from the existing data including images.

As concluding remarks, we address outlook for the metasurface FL biosensors in the near future. In our view, one of the best practices is a scheme as shown in Figure 2B, where AI can contribute to the analysis of big data collected using the productive biosensors. In principle, AI can identify correlations in big data without any preconception and prejudice. To realize this best practice, scientifically reliable, precise big data should be acquired. The metasurface FL biosensors meet this requirement and can function as productive sensing platforms, which are constructed by simple, cost-effective elements, such as CCD cameras, and associated with an application for automated data analysis and collection. This usage of AI has not been emphasized, probably because the productive biosensing systems have not yet become widespread. In the near future, this usage will be one of the standards.

Very recently, this type of AI usage, such as Figure 2B, has been recognized worldwide. The Nobel prize in chemistry 2024 was presented in part to the AI development for computational protein design (Nobel, 2024). Computational molecular dynamics produced big data, which are based on a firm scientific base, were analyzed using AI to explore candidates for new medicines. Just as the computational method served as a productive data generator, the metasurface FL biosensors can function as productive data-taking instruments for opening the next-generation healthcare services.

## Data Availability

The original contributions presented in the study are included in the article/Supplementary Material, further inquiries can be directed to the corresponding author.
