# Supplementary material for: Productive biosensing techniques empowered by all-dielectric metasurfaces
Source: Front Bioeng Biotechnol. 2025 Jan 9;12:1484638. doi: 10.3389/fbioe.2024.1484638 (PMC11770831; doi:10.3389/fbioe.2024.1484638)
Supplement: Supplementary file 1 [file DataSheet1.pdf]

Supplementary Material

1 TABLE

Table S1 lists biosensing techniques appeared in Figure 1A, and their features are specified and compared to each other. Abbriviations stand for as follows:
SDS-PAGE: sodium-dodecyl-sulfate poly-acrylamide gel electrophoresis
ELISA: enzyme-linked immunosorbent assay
qPCR: quantitative polymerase chain reaction

Table S1. Detection methods and their features. Typical detection range is expressed in the units of molar (M), which is mol/L. Precision is classified by setting the current commercial standards, such as ELISA and qPCR, to be standard. Productivity denotes capability of acquiring massive; in this context, the commercial standards are set to standard. #RNA detection is also possible by incorporating reverse transcription procedure.

Method	Target	Typical detection range	Precision	Productivity
SDS-PAGE	Protein	μM–sub nM	Low	Medium
Resonance shift	Protein	μM–pM	Standard	Standard
ELISA	Protein	nM–pM	Standard	Standard
Digital ELISA	Protein	nM–fM	High	Low
Metasurfaces	Protein	nM–fM	High	High
qPCR	DNA#	nM–fM	Standard	Standard
Digital PCR	DNA#	nM–aM	High	Low
Metasurfaces	DNA#	nM–sub aM	Ulrahigh	High
